# The Efficacy and Safety of Linezolid and Glycopeptides in the Treatment of *Staphylococcus aureus* Infections

**DOI:** 10.1371/journal.pone.0058240

**Published:** 2013-03-06

**Authors:** Jinjian Fu, Xiaohua Ye, Cha Chen, Sidong Chen

**Affiliations:** 1 Department of Epidemiology, School of Public Health and Tropical Medicine, Southern Medical University, Guangzhou, Guangdong, China; 2 Guangdong Key Laboratory of Molecular Epidemiology, Department of Epidemiology and Biostatistics, School of Public Health, Guangdong Pharmaceutical University, Guangzhou, Guangdong, China; 3 Department of Laboratory Medicine, Liuzhou Municipal Maternity and Child Healthcare Hospital, Liuzhou, Guangxi, China; 4 Department of Laboratory Medicine, Guangzhou High Education Mega Centre Hospital, Branch of Guangdong Provincial Hospital of Traditional Chinese Medicine, Guangzhou, Guangdong, China; University of Cambridge, United Kingdom

## Abstract

To assess the effectiveness and safety of linezolid in comparison with glycopeptides (vancomycin and teicoplanin) for the treatment of *Staphylococcus aureus* infections, we conducted a meta-analysis of relevant randomized controlled trials. A thorough search of Pubmed and other databases was performed. Thirteen trials on 3863 clinically assessed patients were included. Linezolid was slightly more effective than glycopeptides in the intent-to-treat population (odds ratio [OR], 1.05; 95% confidence interval [CI], 1.01–1.10), was more effective in clinically assessed patients (OR 95% CI: 1.38, 1.17–1.64) and in all microbiologically assessed patients (OR 95% CI: 1.38, 1.15–1.65). Linezolid was associated with better treatment in skin and soft-tissue infections (SSTIs) patients (OR 95% CI: 1.61, 1.22–2.12), but not in bacteraemia (OR 95% CI: 1.24, 0.78–1.97) or pneumonia (OR 95% CI: 1.25, 0.97–1.60) patients. No difference of mortality between linezolid and glycopeptides was seen in the pooled trials (OR 95% CI: 0.98, 0.83–1.15). While linezolid was associated with more haematological (OR 95% CI: 2.23, 1.07–4.65) and gastrointestinal events (OR 95% CI: 2.34, 1.53–3.59), a significantly fewer events of skin adverse effects (OR 95% CI: 0.27, 0.16–0.46) and nephrotoxicity (OR 95% CI: 0.45, 0.28–0.72) were recorded in linezolid. Based on the analysis of the pooled data of randomized control trials, linezolid should be a better choice for treatment of patients with *S. aureus* infections, especially in SSTIs patients than glycopeptides. However, when physicians choose to use linezolid, risk of haematological and gastrointestinal events should be taken into account according to the characteristics of the specific patient populations.

## Introduction

*Staphylococcus aureus*, especially methicillin-resistant *S. aureus* (MRSA) represents a predominant pathogen associated with serious nosocomial and community-acquired infections, including pneumonia, bacteraemia, and complicated skin and soft tissue infections [1]–[4]. Recent data indicated that *S. aureus* accounts for 52% of these infections, with MRSA responsible for 24% of staphylococcal infections [5]. In some Asian countries including China, Japan and Korea, more than 60% of gram positive cocci nosocomial infections were caused by MRSA [6]–[8]. In Europe, the overall prevalence of MRSA was 40% to 45%, and in the United States, 30% to 35% [9], [10].

Increasing MRSA infections result in substantial morbidity and mortality, thus increasing the cost of treatment and the use of medical resources [11]. Although glycopeptide antibiotics (e.g., vancomycin and teicoplanin) have long been the standard treatment for serious infections caused by multidrug resistant gram-positive bacteria, there is an increase in resistance to these antibiotics due to emergence and spread of vancomycin-resistant enterococci [12]–[16]. The pitfalls of vancomycin therapy include poor tissue penetration, adverse effects, the need for intravenous access, and increasing minimum inhibitory concentrations (MICs) among staphylococci [17], [18]. With the rising incidence of gram-positive bacterial infections and the global growing trend of antibiotic resistance, new agents with different mechanisms of action are required to counteract drug resistance or cross-resistance for the treatment of gram-positive infections.

The first available oxazolidinone linezolid is an alternative to vancomycin for effective treatment of gram-positive bacterial infections. It inhibits bacterial protein synthesis by blocking formation of the 70S initiation complex [19], [20]. Linezolid has demonstrated excellent tissue penetration [21], equivalent bioavailability between the oral and intravenous formulations [22], and it lacks cross-resistance with current antibiotic therapies due to its unique mechanism of action.

Several randomized controlled trials have compared linezolid to glycopeptides (vancomycin and teicoplanin) for the treatment of gram-positive bacterial infections. Two recent meta-analyses have demonstrated the superior efficacy of linezolid in the treatment of skin and soft tissue infections [23], [24]. Another meta-analysis showed no inferiority of linezolid treated MRSA skin and soft tissue infections [25]. One meta-analysis comparing vancomycin with linezolid detected no difference between two treatments, which seemed to contradict the other meta-analysis postulating that linezolid therapy was associated with higher clinical cure in patients with gram-positive bacterial infections [24], [26].In light of this controversy, further evaluation of linezolid for its efficacy compared to glycopeptides in the treatment of infections caused by known or suspected MRSA is important. Given the fact that several randomized controlled trials have been performed, and data have become available, we performed a meta-analysis with the goal to study the effectiveness and safety of linezolid in comparison with glycopeptide antibiotics in the treatment of infections caused by known or suspected MRSA.

**Table 1:** Main characteristics of the randomized controlled trials included in the meta-analysis.

Study group	RCT design	District	Patients with infection type	Patient age(M±S.D.)		Enrolled population	Intention to treat	Jadad score
				linezolid	comparator			
Wunderink-2012	Double-blind,	–	Bacteraemia,	60.7±18.0	61.6±17.7	1184	597vs587	5
	multicentre		Pneumonia					
Itani-2010	Multicentre	USA, Europe, Latin America,	cSSTI	49.7	49.4	1077	537vs515	2
		South Africa, Malaysia, Singapore						
Wilcox-2009	Multicentre	Europe, USA	SSTI, Bacteraemia	53.7±18.1	53.8±17.6	739	363vs363	2
Wunderink-2008	Multicentre	USA, Puerto Rico	Pneumonia	55.7±20.5	54.9±19.2	149	74vs72	2
Lin-2008	Double-blind, multicentre	China	SSTI, Pneumonia	56.3±16.7	59.6±13.3	144	71vs71	4
Kohno-2007	Multicentre	Japan	SSTI, Pneumonia	68.4±16.4	67.5±16.3	154	100vs51	2
Weigelt-2005	Multicentre	USA	cSSTI	52±18	52±18	1200	592vs588	2
Cepeda-2004	Double-blind,	UK	Bacteraemia,	≥16 yr	≥16 yr	100vs104	100vs102	5
	multicentre		Pneumonia					
Wilcox-2004	Multicentre	Europe, Latin America	SSTI, Bacteraemia, Pneumonia	53±20	55±19	219vs219	215vs215	3
Kaplan-2003	Multicentre	USA, Latin America	SSTI, Bacteraemia,	2.2±3.2	2.9±3.1	219vs102	215vs101	2
			Pneumonia					
Wunderink-2003	Double-blind,	North, South America, Europe,	Pneumonia	63.1±19.1	61.9±19.3	623	321vs302	4
	multicentre	Israel, South Africa, Australia						
Stevens-2002	Single-blind,	North America, Europe,	SSTI, Bacteraemia,	63.9±16.1	59.8±20.2	240vs220	240vs220	2
	Multicentre	Latin America, Asia	Pneumonia					
Rubinstein-2001	Double-blind,	North, South America, Europe,	Pneumonia	62.8±18.0	61.3±18.7	402	203vs193	3
	multicentre	Israel, South Africa, Australia						

cSSTI, complicated skin and soft-tissue infection; SSTI, skin and soft-tissue infection; yr, year.

## Methods

### Data Sources

The meta-analysis was conducted following the PRISMA guidelines [27]. An extensive search of PubMed (January 1, 1995, to September 15, 2012), Current Contents, Embase, Scopus, Cochrane Central Register of Trials database was performed to identify relevant trials. Search terms included: linezolid; oxazolidinone; vancomycin; teicoplanin; glycopeptides; skin and soft tissue; pneumonia; bacteraemia; gram-positive cocci; *S. aureus*; MRSA; enterococcus; infections; randomized; prospective. Published abstracts from major international conferences (CHEST, American Thoracic Society, Infectious Diseases Society of North America) were also searched but not included in the meta-analysis. Two reviewers (JJ Fu and XH Ye) independently searched the literature and examined relevant trials for further assessment of data on effectiveness and toxicity. Any disagreements were resolved by consensus.

### Inclusion Criteria for Trials

A study was considered eligible if it was a randomized controlled clinical trial, if it studied the role of linezolid in comparison with a glycopeptide in the treatment of infectious caused by *S. aureus*, and if it assessed the effectiveness, toxicity, or mortality of both therapeutic regimens. A study would be excluded if it was an experimental trial or if it focused on pharmacokinetic or pharmacodynamic variables. Additional antimicrobial agents (those with effectiveness against gram-negative rods involved in polymicrobial infections) could be used in the analysis.

### Data Extraction

The following data were extracted from each study: authors, publication year, study design, study district, patients with infection type, mean age, patient population (intention to treat [ITT], clinical evaluation [CE] and microbiological evaluation [ME]), sample size, antimicrobial agents and doses used, mean treatment duration, clinical outcome, microbiological eradication, adverse events, patients withdrawn because of adverse effects and mortality. The ITT population consisted of all randomized patients who received at least one dose of study medication. The CE population included patients who fulfilled all inclusion and exclusion criteria in the individual trial, who had complete follow-up and for whom data on treatment outcomes were available. The ME population was a subset of the CE population who had microbiologically documented infections.

According to a modified Jadad score [28], a quality review of each trial was performed to include details of randomization, generation of random numbers, details of the double blinding procedure, information on withdrawals, and allocation concealment. One point was awarded for the specification of each criterion, with a maximum score of 5. Scores of 3 or more points were high-quality trials, whereas those with 2 or fewer points were low-quality trials.

### Efficacy and Safety Definitions

Treatment success included clinical cure and microbiological evaluation. Clinical cure was assessed in all patients who had complete follow-up and separated in patients with SSTIs, bacteraemia and pneumonia. Microbiological assessment and documented eradication of *S. aureus* and MRSA were secondary outcomes. Mortality was defined as all-cause deaths during treatment and follow-up period. Haematological effects included leucopenia, thrombocytopenia, anemia and haemolysis. Gastrointestinal effects included dyspepsia, nausea, vomiting, liver disease, pancreatitis, diarrhea and loose stools. Skin effects included rash, pruritus and red man syndrome. Nephrotoxicity included acute kidney failure and renal impairment.

### Statistical Analysis

Statistical analyses were done with STATA version 10.0. The data were pooled by using the Mantel-Haenszel fixed-effects model (FEM) [29] and the DerSimonian and Laird random-effects model (REM) [30]. For all analyses, results from the FEM were presented only when there was no heterogeneity between trials, otherwise results from the REM are presented. Publication bias were examined by funnel plot and further accessed by Egger’s test, with *P*<0.10 indicating potential bias [31].

## Results

### Main Characteristics of the Pooled Trials

**Figure 1:**
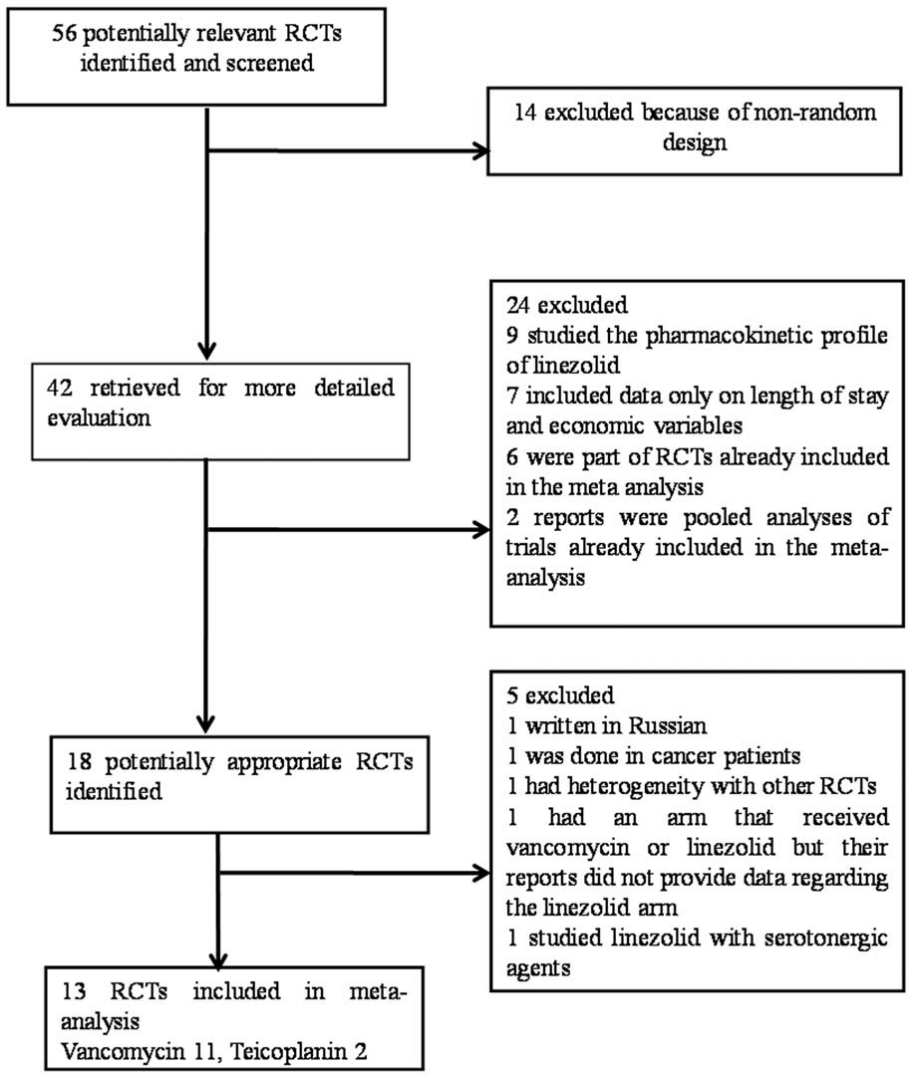
Flow diagram of the randomized controlled trials (RCTs) for meta-analysis.

The flow diagram for selection of trials used in the final analysis is shown in Figure 1. By reading the abstracts and using our inclusion/exclusion criteria, forty-two trials were selected for further investigation. Twenty-nine reports of trials [32]–[60] were excluded for the reasons noted in Figure 1. Finally, thirteen trials met the inclusion criteria of our study [61]–[73], yielding a total of 3,863 patients (Table 1).

### Selected Randomized Controlled Trials

The main characteristics of the analyzed trials are given in Table 1. The mean quality score of the included 13 trials was 2.9 (range 2–5), 6 trials (46%) were high quality (score≥3). All enrolled patients had a presumed or documented infection caused by *S. aureus*. Patients with SSTIs, bacteraemia or pneumonia were analyzed further. Administration of any antibiotics effective against *S. aureus* in the previous 24–48 h, including the study antibiotics, was not allowed in all the trials.

### Treatment Success in Clinically Evaluable (CE) Populations

**Figure 2:**
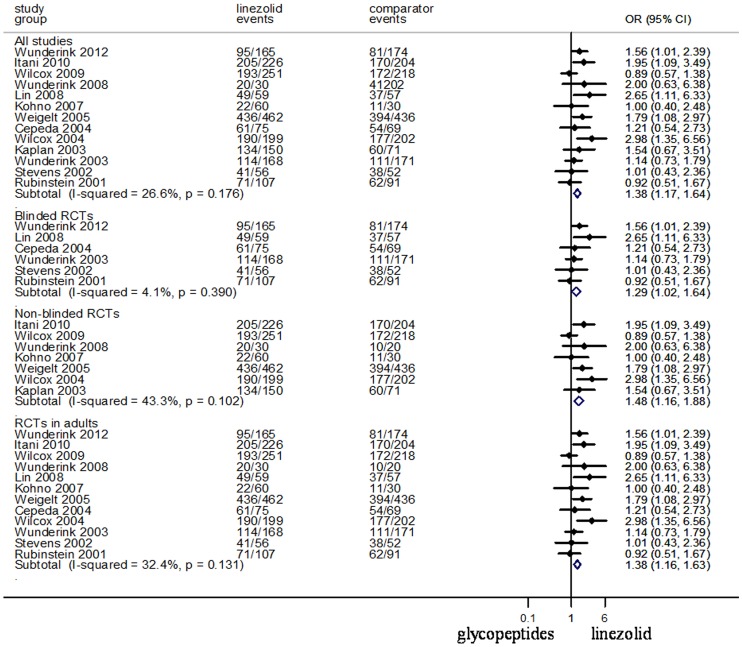
Meta-analyses of treatment success for clinically assessed patients. Test of all studies for overall effect: Z = 3.65 *P* = 0.000; test of blinded RCTs for overall effect: Z = 2.11 *P* = 0.035; test of Non-blinded RCTs for overall effect: Z = 3.03 *P* = 0.002; test of RCTs in adults for overall effect: Z = 3.64 *P* = 0.000.

The primary clinical outcomes that were included in the meta-analysis are shown in Figure 2. Data on treatment success of the regimen for ITT and CE populations was reported in eight and thirteen of the trials, respectively. Linezolid was slightly more effective than glycopeptides in the ITT population (N = 3130, OR 95%CI: 1.05, 1.01–1.10). Success of empirical treatment in clinically assessed patients was achieved in 80.2% of linezolid-treated patients and in 76.3% of glycopeptides-treated patients. Linezolid was also more effective than glycopeptides in the CE population (N = 3863, OR 95%CI: 1.38, 1.17–1.64). When data from blinded RCTs only were analyzed, treatment with linezolid was associated with better treatment success in CE populations than glycopeptides (N = 1244, OR 95%CI: 1.29, 1.02–1.64). When combined with the non-blinded trials, linezolid treatment was found to be more effective than glycopeptides (N = 2559, OR 95%CI: 1.48, 1.16–1.88). The same was true for clinically assessed adult populations (N = 3582, OR 95%CI: 1.38, 1.16–1.63).

**Figure 3:**
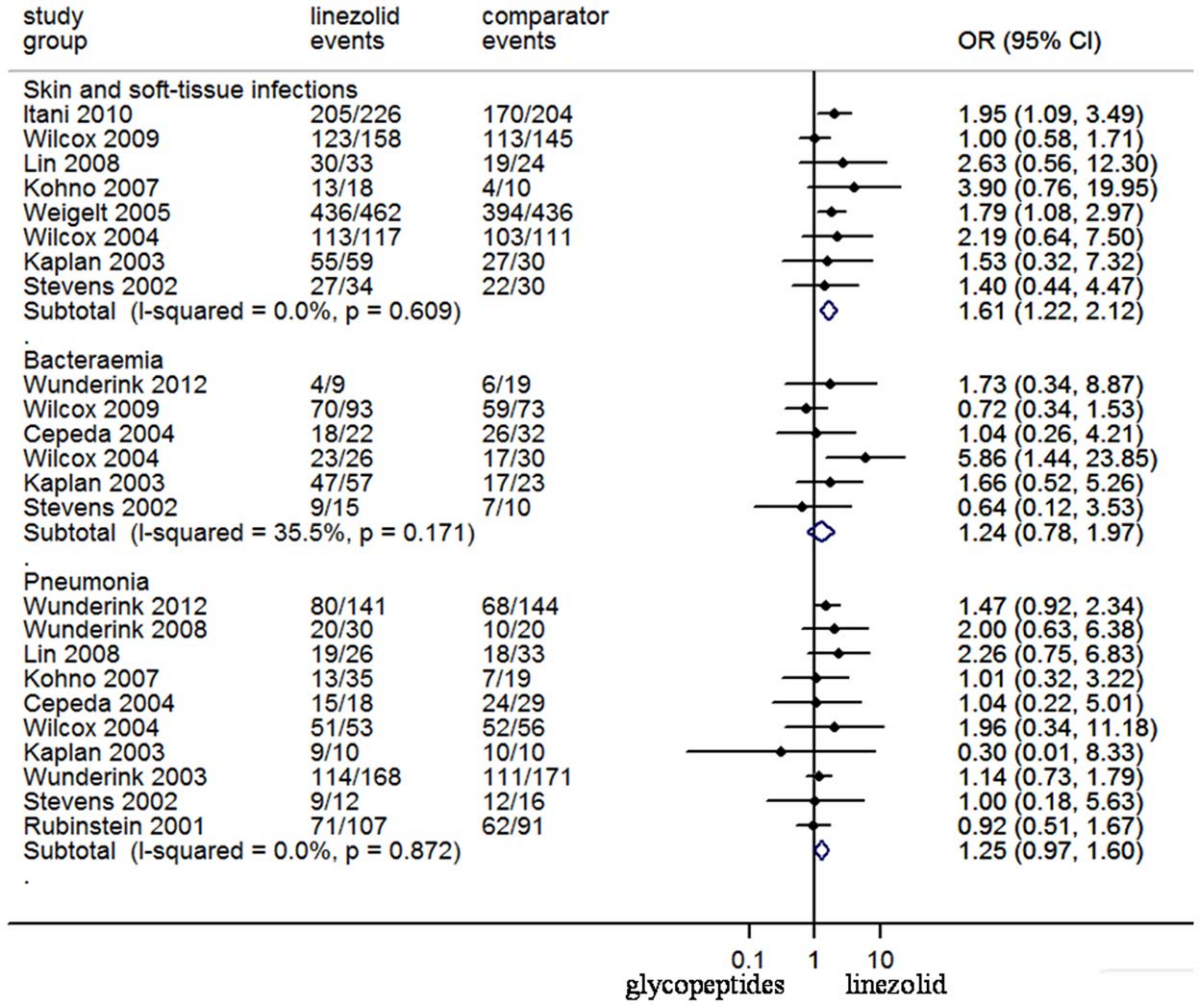
Meta-analyses of treatment success for clinically assessed patients with skin and soft-tissue infections, bacteraemia, and pneumonia. Test of SSTI for overall effect: Z = 3.37 *P* = 0.001; test of bacteraemia for overall effect: Z = 0.91 *P* = 0.364; test of pneumonia for overall effect: Z = 1.73 *P* = .083.

The pooled data in the meta-analysis for SSTIs, bacteraemia and pneumonia were summarized in Figure 3. Success of the empirical treatment was achieved in 90.5% of linezolid-treated patients and in 86.1% of glycopeptides-treated patients in 8 RCTs that reported data on SSTIs. Empirical treatment of patients with SSTIs with linezolid was associated with significantly better success than glycopeptides (N = 2097, OR 95%CI: 1.61, 1.22–2.12).

Six trials reporting outcomes for patients with bacteraemia were available with empirical treatment success occurring in 171 of 222 (77.0%) linezolid-treated patients and in 132 of 187 (70.6%) glycopeptides-treated patients. There was no significant difference in treatment success for bacteraemia between linezolid and glycopeptides (OR 95%CI: 1.24, 0.78–1.97). The effectiveness outcomes for pneumonia were available from 10 reported RCTs. Empirical treatment success occurred in 401 of 600 (66.8%) linezolid-treated patients and in 374 of 589 (63.5%) glycopeptides-treated patients. There was no difference in treatment success for pneumonia between linezolid and glycopeptides (OR 95%CI: 1.25, 0.97–1.60).

### Treatment Success in Microbiologically Evaluable (ME) Populations

**Figure 4:**
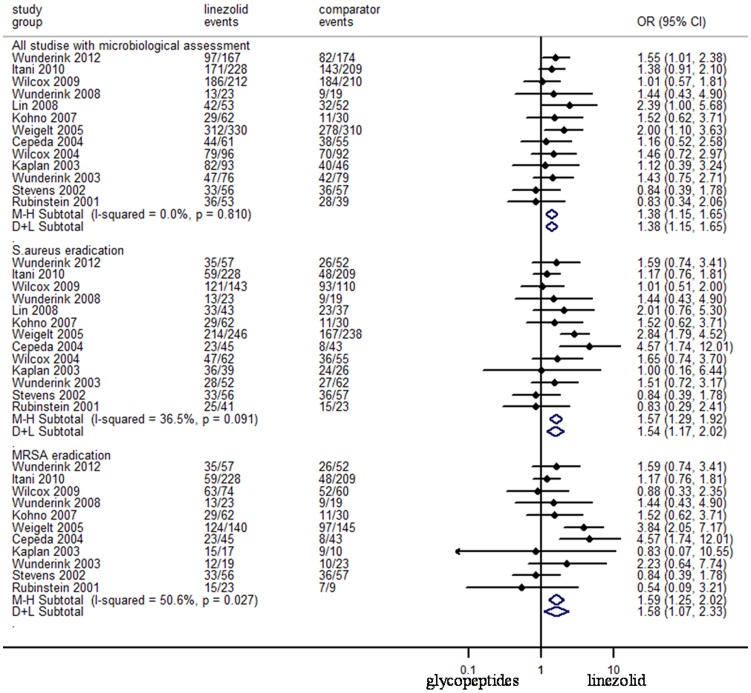
Meta-analyses of treatment success for microbiologically assessed patients. Test of all studies with microbiological assessment for overall effect: Z = 3.46 *P* = 0.001; test of *S.aureus* eradication for overall effect: Z = 4.43 *P* = 0.000; test of MRSA eradication for overall effect: Z = 3.78 *P* = 0.000.

All thirteen RCTs included in the meta-analysis reported data on microbiologically assessed patients was shown in Figure 4. Empirical treatment of gram-positive infections with linezolid was associated with better treatment success than glycopeptides (N = 2882, OR 95%CI: 1.38, 1.15–1.65). Empirical treatment with linezolid was associated with better eradication rates for *S. aureus* and MRSA in comparison with glycopeptides (N = 2058, OR 95%CI: 1.54, 1.17–2.02), (N = 1401, OR 95%CI: 1.58, 1.07–2.33), respectively.

### Adverse Effects

**Figure 5:**
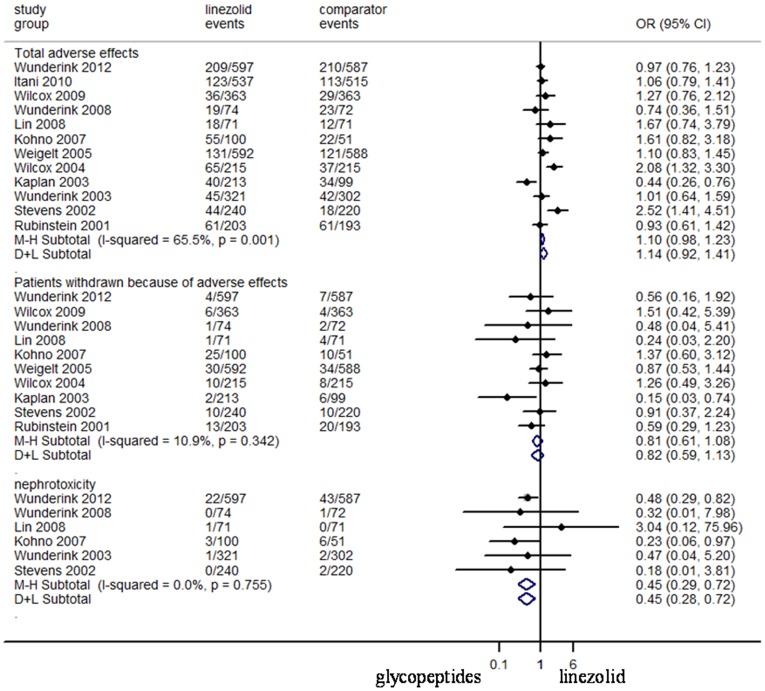
Meta-analyses of adverse effects related to studied regimens. Test of total adverse effects for overall effect: Z = 1.58 *P* = 0.113; test of patients withdrawn because of adverse effects for overall effect: Z = 1.41 *P* = 0.159; test of nephrotoxicity for overall effect: Z = 3.36 *P* = 0.001.

**Figure 6:**
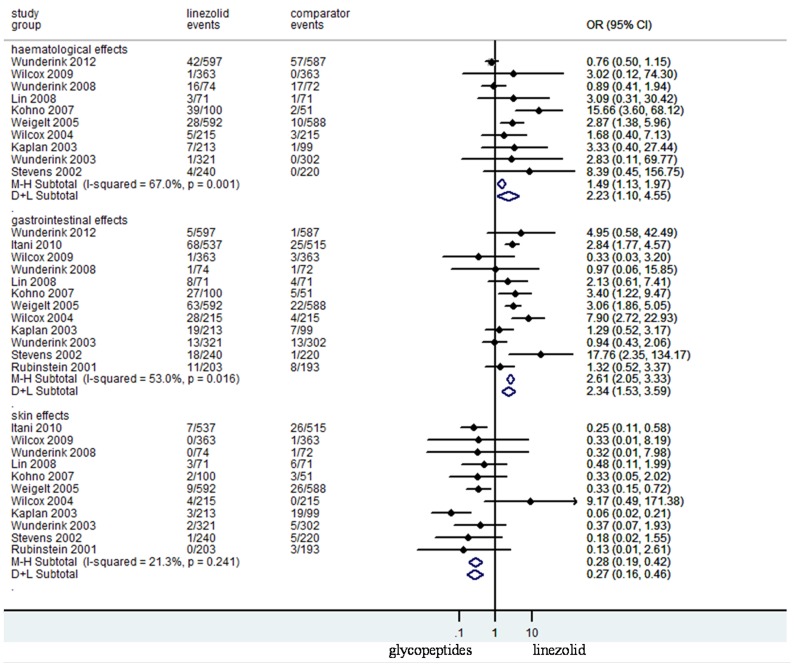
Meta-analyses of haematological, gastrointestinal, and skin adverse effects related to studied regimens. Test of haematological adverse effects for overall effect: Z = 2.56 *P* = 0.010; test of gastrointestinal adverse effects for overall effect: Z = 7.74 *P* = 0.000; test of skin system adverse effects for overall effect: Z = 6.27 *P* = 0.000.

**Figure 7:**
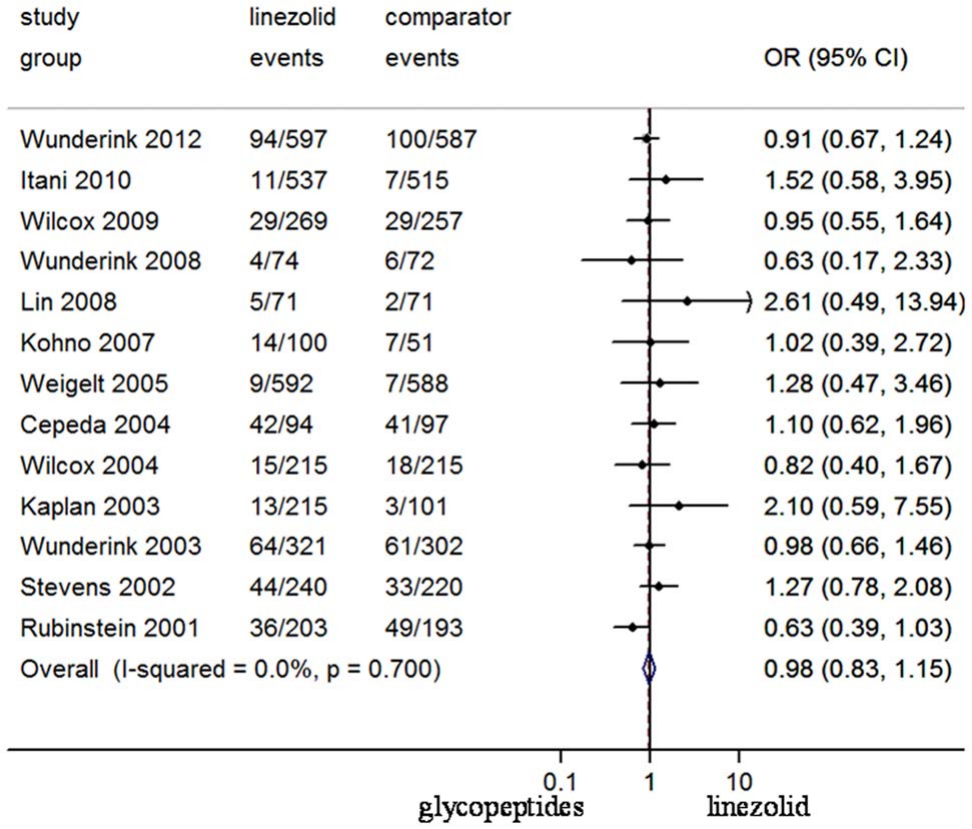
Meta-analysis of mortality in this pooled data. Test of mortality for overall effect: Z = 0.29 *P* = 0.771.

Although the drop-out rate was high in some RCTs (Figure 5), there was no significant difference between the treatment groups in the proportion of patients who were withdrawn from RCTs due to adverse effects (N = 5129, OR 95%CI: 0.82, 0.59–1.13). Data on adverse effects possibly related to the study regimens were reported in all trials. There was no difference between the study medications of total adverse effects (N = 6802, OR 95%CI: 1.14, 0.92–1.41) and patients withdrawn from trials (N = 5127, OR 95%CI: 0.82, 0.59–1.13). Linezolid was associated with more haematological adverse effects (N = 5354, OR 95%CI: 2.23, 1.10–4.55), and gastrointestinal adverse effects (N = 6802, OR 95%CI: 2.34, 1.53–3.59), respectively (Figure 6). Meanwhile, significantly less episodes of skin adverse effects (N = 5018, OR 95%CI: 0.27, 0.16–0.46), and nephrotoxicity (N = 2706, OR 95%CI: 0.45, 0.28–0.72) were reported in linezolid-treated patients (Figures 5 and 6). The mortality risk between linezolid and glycopeptides (N = 6797, OR 95%CI: 0.98, 0.83–1.15) was not different (Figure 7).

### Publication Bias

Visual inspection of funnel plot and statistical tests suggested no indication of publication bias for studies on CE population (Figure S1; Begg’s test *P* = 0.428, Egger’s test *P* = 0.318), patients with SSTI, bacteraemia and pneumonia (Figure S1; Begg’s test *P* = 1.000, Egger’s test *P* = 0.416), ME population (Figure S1; Begg’s test *P* = 0.897, Egger’s test *P* = 0.563), all related adverse effects (Figure S1; Begg’s test *P* = 0.960, Egger’s test *P* = 0.796), and mortality (Figure S1; Begg’s test *P* = 0.127, Egger’s test *P* = 0.136).

## Discussion

This pooled meta-analysis of randomized controlled trials for suspected *S. aureus* infections suggested that linezolid was significantly more effective for the treatment of all patients (those with SSTIs, bacteraemia and pneumonia) with *S. aureus* infections than glycopeptides. The data point toward a significantly higher effectiveness of linezolid when compared with glycopeptides in both blinded and non-blinded RCTs. Empirical linezolid treatment was associated with better treatment success in microbiologically assessed patients, and both the patient populations having either *S. aureus* or MRSA infection.

Empirical linezolid treatment was superior to glycopeptides with respect to patients with SSTIs. However, it should be noted that the comparative effectiveness of linezolid and glycopeptides relies mainly on open-label trials [62], [63], [66], [67], [69], [70]. When excluding the non-blinded RCTs, the remaining two blinded RCTs [65], [72] showed that glycopeptides were noninferior to linezolid for patients with SSTIs (OR 95%CI: 1.76, 0.70–4.43). On the other hand, the reported good penetration of linezolid into skin, and the availability of an oral formulation were important factors shown in several studies that may partly explain the higher efficacy of linezolid for the treatment of SSTIs, despite its higher acquisition cost.

The results of this meta-analysis suggest that linezolid is as effective as glycopeptides for the treatment of patients with bacteraemia and pneumonia due to *S. aureus* infections. When it comes to the treatment of patients with bacteraemia infections, several issues must be addressed. Firstly, the available evidence for the effectiveness of comparator antibiotics for the treatment of bacteraemia patients is limited. Secondly, the absolute number of some reported bacteraemia infection cases was small, which would lead to some heterogeneity between RCTs when compared with the treatment outcomes. It is noteworthy that a recently pooled meta-analysis also concluded that vancomycin was noninferior to linezolid for the treatment of *S. aureus* bacteraemia [24], which was contradictory to the other meta-analysis stating that linezolid therapy was associated with higher clinical cure in patients with gram-positive bacteraemia infections [23]. Some limitations of the latter meta-analysis should be known. Trials of the evaluation of bacteraemia infections included in this study were published in 2002–2004, only 5 trials were pooled, one of them focusing on the effectiveness of linezolid in comparison with β-lactam for the treatment of bacteraemia patients [23]. When this study was excluded, linezolid was not less effective than glycopeptide antibiotics in this patient population (OR 95%CI: 1.15, 0.98–1.35).

No difference in treatment of pneumonia infections was noticed in the analysis of all pooled trials, although an updated trial published recently showed that linezolid was more effective than vancomycin for the treatment of MRSA nosocomial pneumonia [61]. Similar to our results, all of the prior meta-analyses [23], [24], [26], [75], [76] demonstrated that linezolid was clinically as effective as glycopeptides for the treatment of pneumonia. These findings can be explained by various assumptions. Although one report [59] hypothesized that superior drug concentrations of linezolid in the lung would be a potential mechanism which benefits for treatment of MRSA pneumonia infection, some confounding factors such as protein binding, decreased alveolar macrophage concentrations through cell lysis and antibiotic diffusibility [60] could alter drug concentration and potentially lead to confusing results. Secondly, it is not known if subtherapeutic pulmonary drug concentration was higher in patients with pneumonia in linezolid arm compared with glycopeptides arm because of the lack of reporting of the pooled trials in our study. Thirdly, the MIC of vancomycin was not regularly monitored in the pooled trials. It is noted that high MICs are a known risk factor for vancomycin failure in MRSA bacteraemia and pneumonia [79], [80]. A better choice of linezolid would likely be taken when vancomycin MICs are >1 µg/ml as supported by consensus guidelines [81]. Besides, a large number of included patients in the pooled trials also received additional antibiotics for the treatment of gram-negative bacterial infections, which may have contributed to the increased effectiveness of studied antibiotics in some cases.

The currently available data indicates that teicoplanin is not inferior to linezolid with regard to treatment efficacy for pneumonia. However, no consistence results compared linezolid with teicoplanin in treatment of *S. aureus* bacteraemia is found. The lack of published data regarding the effectiveness of linezolid compared with teicoplanin for treatment of *S. aureus* infection is remarkable and maybe the explanation of the contradict results, although there is some evidence suggest that linezolid has a greater probability of attaining its requisite pharmacodynamic target than teicoplanin against *S. aureus* infection [82]. Because of the limited number of cases within each comparative group, caution should be taken in interpreting results. Further larger randomized controlled trials are required to confirm the efficacy and safety of linezolid and teicoplanin in the treatment of *S. aureus* infection.

No difference in mortality was noticed in the pooled trials. Of note, 7 out of 13 studies were unblinded [62]–[64], [66], [67], [70], [72]. No significant evidence of publication bias for studies on mortality was observed in both blinded and open-label pooled trials. The results were inconsistent with the latest published meta-analysis [26] which considered a higher potential for bias would occur in the open-label trials.

Linezolid was associated with similar rates of adverse events with comparator regimens. The risk of haematological and gastrointestinal effects was approximately doubled with linezolid in comparison with studied medications. On the other hand, glycopeptides were associated with more episodes of skin adverse effects and nephrotoxicity than linezolid, although the event rates for nephrotoxicity and skin adverse effects were lower than those for haematological and gastrointestinal effects. Nephrotoxicity was mainly seen with vancomycin, which was consistent with recent studies [24], [26].

The present meta-analysis has some limitations. There are some missing data from original reports which the authors could not receive from the investigators performing the trials, and thus may have introduced bias to the reported outcomes of effectiveness. Seven open-label trials meeting the criteria of randomization were included in the pooled data, the lower methodological quality may have introduced bias to the reported outcomes, although no publication bias of clinical, microbiological and survival outcomes were seen in this study. Furthermore, vancomycin serum concentrations that were not routinely monitored in several trials might have contributed to lower treatment success of the regimen, thus influencing the outcomes in favor of linezolid. Finally, a large proportion of trials did not provide the data of patients with proven *S. aureus* infections, which may have influenced the treatment outcomes.

This meta-analysis shows that linezolid is associated with better clinical and microbiological outcomes than glycopeptides for the treatment of *S. aureus* infections. Moreover, the data shows that linezolid is more effective than glycopeptides for the treatment of SSTIs. Our data did not detect superiority of linezolid over glycopeptides for the treatment of bacteraemia or pneumonia in terms of clinical cure. Linezolid was associated with more haematological and gastrointestinal events. Compared to linezolid, glycopeptides showed a significant increase in the risk of skin adverse effects and nephrotoxicity. Vancomycin has been assumed to be the first choice of treatment for patients with *S. aureus* infections, especially with long term MRSA infections. It is inspiring that an alternative, which is more effective, or at least equally effective in some cases, is available for patients with *S. aureus* infections. However, the higher risk of haematological and gastrointestinal events should be taken into account and may limit the use of linezolid according to the characteristics of the individual patient.

## Supporting Information

Figure S1 **Begg’s funnel plot with 95% confidence limits to detect publication bias. Each point represents a separate study for the indicated association.**
(DOC)

Table S1 **PRISMA checklist of this meta-analysis.**
(DOC)
