# Supplementary material for: The Efficacy and Safety of Linezolid and Glycopeptides in the Treatment of Staphylococcus aureus Infections
Source: PLoS One. 2013 Mar 6;8(3):e58240. doi: 10.1371/journal.pone.0058240 (PMC3590119; doi:10.1371/journal.pone.0058240)
Supplement: Figure S1 — Begg’s funnel plot with 95% confidence limits to detect publication bias. Each point represents a separate study for the indicated association. (DOC) [file pone.0058240.s001.doc]

Figure S1


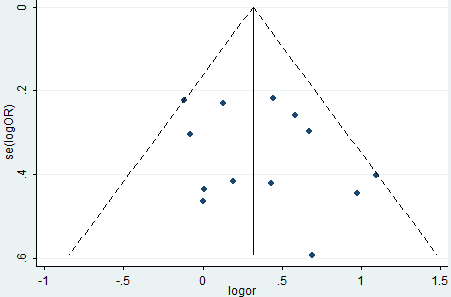

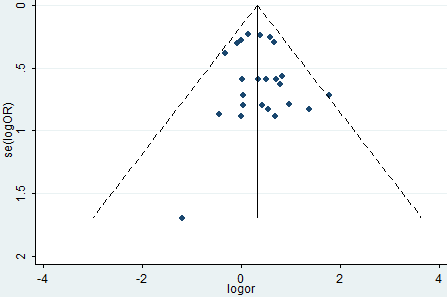


CE population patients with SSTI, bacteraemia and pneumonia


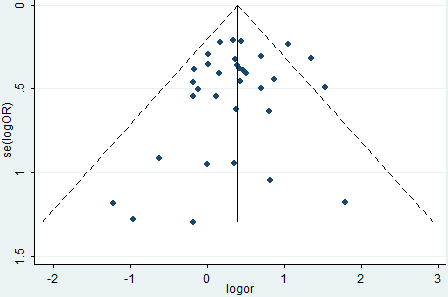

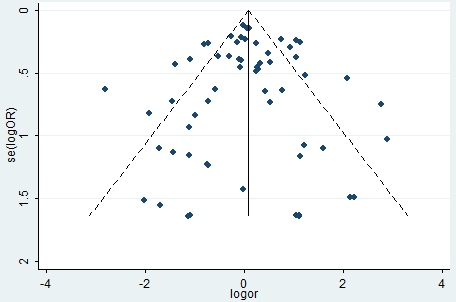


ME population all related adverse effects


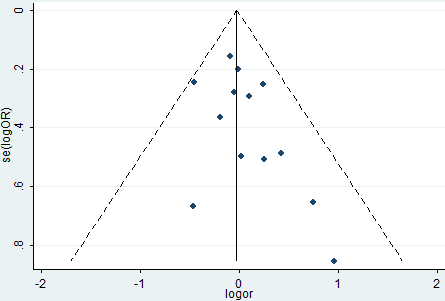


mortality
